# Can Neurocognitive Function Predict Lower Extremity Injuries in Male Collegiate Athletes?

**DOI:** 10.3390/ijerph17239061

**Published:** 2020-12-04

**Authors:** Sunghe Ha, Hee Seong Jeong, Sang-Kyoon Park, Sae Yong Lee

**Affiliations:** 1 Department of Physical Education, College of Sciences in Education, Yonsei University, Seoul 03722, Korea; sunghe.ha@yonsei.ac.kr (S.H.); hsj@yonsei.ac.kr (H.S.J.); 2 International Olympic Committee Research Centre Korea, Yonsei University, Seoul 03722, Korea; 3 School of Physical Education, Korea National Sport University, Seoul 05541, Korea; 4 Institute of Convergence Science, Yonsei University, Seoul 03722, Korea

**Keywords:** lower limb, men, non-net sports, prevention, screening

## Abstract

The purpose of this study is to demonstrate whether neurocognitive evaluation can confirm the association between neurocognitive level and postural control and to analyze the relationship between neurocognitive level and acute musculoskeletal injury in male non-net sports athletes. Seventy-seven male non-net sports athletes participated in this study. The Standardized Assessment of Concussion (SAC), Landing Error Scoring System (LESS), Balance Error Scoring System (BESS), and Star Excursion Balance Test (SEBT) were used for testing; we collected data related to injury history for six months after testing. Pearson’s correlation analysis, logistic regression, and the independent sample *t*-test were used for statistical analysis. The correlation between SAC and SEBT results was weak to moderate (*p* < 0.05). Eleven of the seventy-seven participants experienced acute lower limb injuries. SAC, LESS, BESS, and SEBT results have no effect on the occurrence of acute lower extremity injuries (*p* > 0.05) and were not statistically different between the injured and non-injured groups (*p* > 0.05). Therefore, using the SAC score alone to determine the risk factor of lower extremity injuries, except in the use of assessment after a concussion, should be cautioned against.

## 1. Introduction

The occurrence of concussion among male elite athletes participating in contact sports is reported to be higher than that of women, accounting for approximately 66%–76% of the overall incidence [1,2,3,4]. In particular, the incidence of concussion was highest in adolescents and young adults [4]. Because of the nature of non-net sports such as contact with other players or objects, shocks to the head, neck, and upper body are frequent, and the accumulation of these shocks causes serious problems, for instance, concussions [5] and the possibility of cognitive decline [6]. According to a recent study analyzing the causal relationship between cognition and musculoskeletal injury, musculoskeletal injury may occur at a high level when participating in sports if the level of cognition is low or lowered owing to concussion [7,8,9]. Elite athletes who returned to sports after a concussion showed that the likelihood of acute lower musculoskeletal injury was increased compared with non-injured athletes [10,11].

Musculoskeletal injuries cause joint instability, recurrent injury, and other site injuries, as well as premature degenerative osteoarthritis [12] and accelerated retirement [13]. Various field studies have been reported to reduce the incidence of musculoskeletal injury, but several injuries have still been reported [14,15,16]. Along with current approaches to reduce sports injuries, new and efficient methods are needed in the field of sports. Most studies have suggested a link between cognition and sports injuries using expensive equipment such as computerized neurocognitive testing [17,18], magnetic response imaging [19], and electroencephalography [20]. However, a tool that can efficiently assess risk factors of musculoskeletal injury to athletes through a paper-and-pencil method and that is less expensive and requires less time than the computerized methods currently available is needed.

The Standardized Assessment of Concussion (SAC) was designed to quickly apply, observe, and evaluate the orientation, immediate memory, concentration, and delayed memory of an athlete with a head injury [21]. A reduction in SAC score according to head impact can be said to be a neurocognitive dysfunction [22]. Neurocognitive screening, the third item of the Sport Concussion Assessment Tool-Fifth Edition, is composed of SAC and is the most used method in the field [23,24]. However, its application as an assessment tool for neurocognitive impairment with regard to the accumulation of repetitive shocks during participation in non-net sports is insufficient. Repeated impact on the head results in decreased neurocognitive function, resulting in musculoskeletal injuries when participating in sports.

Due to the notion supported from neuroimage studies suggesting that the motor and neurocognitive process possesses the common neural pathway and resources [25,26,27], the studies trying to identify an association between neurocognitive function and postural control has been conducted [28,29]. This can explain the possibility of impairment of neuromuscular control if neurocognitive function has been damaged by repetitive impact. The association between neurocognitive function and motor skill may be affected by level of neurocognitive control process (difficulty/complexity of the task). Therefore, different types of field tasks assessing motor skills (e.g., static and dynamic postural control) should be employed to address and evaluate its association with neurocognitive function.

Static and dynamic balances are considered an important aspect of performance and injury risk of the lower extremity in many athletic events. Balance Error Scoring System (BESS), Landing Error Scoring System (LESS), Star Excursion Balance Test (SEBT), etc., used without expensive equipment in a clinical setting are applied to assess static and dynamic postural control capabilities, which are reported to be predictable for ligament sprains of the lower extremity [30,31,32]. These postural control test methods are not only used as baseline tests to monitor players before the season but also as evaluation criteria for returning to sports.

This study aims to verify whether neurocognitive assessment can identify association between neurocognitive level and postural control and analyze the association between the neurocognitive level and the occurrence of acute musculoskeletal injuries in male non-net sports athletes. This study hypothesized that there would be correlations between the neurocognitive evaluation scores and scores of postural control of the lower extremity, and the neurocognitive evaluation scores could predict acute lower limb injuries.

## 2. Methods

### 2.1. Participants

Seventy-seven male elite college players of 14 basketball, 22 rugby, 11 baseball, 15 ice hockey, and 15 soccer players participated in this study (height, 180.0 ± 7.4 cm; body weight, 83.9 ± 15.0 kg; age, 19.7 ± 1.3 y). All selected participants had no history of orthopedic acute injury or concussion in the previous six months, were registered as elite athletes, and participated in training and competition.

### 2.2. Experimental Design

This study was conducted with the approval of the Bioethics Committee of Yonsei University (7001988-201810-HR-465-03). Informed consent of voluntary participation was received from all study participants. After receiving informed consent, neurocognitive examination to detect injury risk of the lower extremity was performed using the screening tool, and follow-up investigation on injury occurrence was conducted.

#### 2.2.1. Standardized Assessment of Concussion

As a noninvasive tool for determining brain dysfunction resulting from to sports concussion, the Korean version of SAC, which was verified for reliability and validity, was used for the neurocognitive evaluation test (conformity, 0.88–1.1; external compatibility, approximately 0.55–1.45; separation index, 4.59; separation reliability, 0.95) [33]. SAC is a form of scoring an answer through a tester’s question and consists of a mental test, an immediate memory test, a concentration test (speaking in reverse order of numbers), a concentration test (subtracting 7 consecutive numbers from 100), and a delayed memory test. The total score for the Korean version is 37, with higher scores equating to better scores.

#### 2.2.2. Postural Control of the Lower Extremity

Two video cameras (HDR-PJ410; Sony, Tokyo, Japan; EOS 800D; Canon, Tokyo, Japan) were used to evaluate the performance of the lower extremities (sampling rate, 60 Hz). Performing action is an evaluation tool for predicting lower limb injury and returning to rehabilitation. LESS, BESS, and SEBT have been used in the sports field.

**Figure 1:**
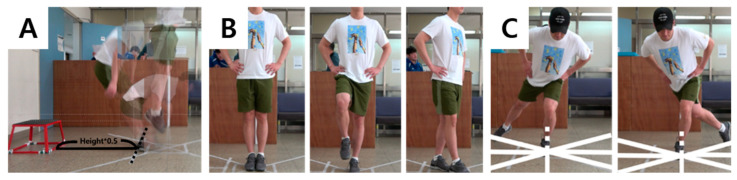
Experimental set-up to detect the injury risk of lower extremities: (**A**) Landing Error Scoring System; (**B**) Balance Error Scoring System; (**C**), Star Excursion Balance Test.

In LESS, when the subject is ready for the motion test on a 30 cm box, the subject jumps both feet lightly and lands at 50% of the height and then immediately performs the maximum vertical jump (Figure 1A). A detailed explanation to perform the task successfully was provided to the participants and practice trials were provided three times. No feedback was provided during the task. If the instructions were followed, it was considered to be successful.

The BESS uses an action in which the subject closes his eyes and puts both hands on the right and left iliac crests and maintains double-leg standing (feet stand together), single-leg standing (on the nondominant leg), and tandem standing (nondominant foot behind the dominant foot) for 20 s each (Figure 1B). The ground condition was carried out on a flat floor and soft board. Participants were provided with a full description of the movement and warm-up, and practices were conducted thrice for each movement. No feedback was provided during the task.

In the SEBT (Figure 1C), the subjects stood on one leg in the center of the grid, with both hands placed on the left and right iliac crests. They were asked, along eight lines drawn at a 45-degree interval, to extend the legs as far as possible and touch the floor lightly with their toe. The direction of eight lines is as follows: anterior (A), anteromedial (AM), medial (M), posteromedial (PM), posterior (P), posterolateral (PL), lateral (L), and anterolateral (AL). If the subject falls off the fixed hand from the iliac crests and fails to stand on one foot or if the fixed footfalls or the foot fails to return to the starting position, it was considered a failure and was conducted again. Participants were provided with detailed instructions to successfully perform the task. A total of three practice tests were performed, and no feedback was provided during the task. The task was considered successful if the participants followed the instructions successfully.

For the follow-up of injury occurrence, the injury investigation form used by the International Olympic Committee was used for six months after neurocognitive examination and motion analysis [34]. The injury was defined as occurring acutely in the musculoskeletal system of the lower extremity, except for progressive onset and chronic pain. The history of injury of some participants who have team trainers was directly recorded by the trainer on the online-injury surveillance system, which was developed by YISSEM based on recommendations of International Olympic Committee, and was collected, and soccer participants without team trainers were contacted individually every two months by the author, with their injuries being recorded.

### 2.3. Data Processing

The SAC calculated the sub-domains and total score, respectively, for orientation, immediate memory, concentration, and delayed memory. 

The LESS scored the error action by observing the initial contact of the ground, the maximum knee flexion, and the overall landing in the sagittal and coronal planes [30]. The lower the error score, the better the landing. The higher the score, the worse the landing (excellent landing, ≤4 points; good landing, 5 points; normal landing, 6 points; wrong landing, >6). There are 17 LESS items for error scoring: (1) the knee flexion angle of <30 degrees at initial contact (IC); (2) thigh in line with the trunk at IC; (3) trunk vertical or in line with the hips at IC; (4) foot landing heel to toe or with a flat foot at IC; (5) center of the patella being medial to the midfoot at IC; (6) trunk lateral flexion at IC; (7) asymmetric initial foot contact; (8) stance width greater than the shoulder width at IC; (9) stance width less than the shoulder width at IC; (10) external rotation of the foot >30 degrees between IC and maximum knee flexion; (11) internal rotation of the foot >30 degrees between IC and maximum knee flexion; (12) knee flexion angle of <45 degrees between IC and maximum knee flexion; (13) thigh not flexing more on the trunk between IC and maximum knee flexion; (14) trunk not flexing more between IC and maximum knee flexion; (15) center of the patella being medial to the great toe during landing; (16) displacement of the trunk, hips, and knees during landing; and (17) overall impression during landing [30]. Items 1–15 receive 1 point each if the above conditions are met. In contrast, item 16 is evaluated as 1 point for average and 2 points for stiff, and item 17 is evaluated as 1 point for average and 2 points for poor [30]. Kinovea (version 0.8.27; Kinovea, https://www.kinovea.org/) software was used to evaluate the knee angle among the items by the same rater (reliability: 0.941). 

The BESS was conducted for 20 s. If an error of operation was observed 5 s before, 10 points were scored [35]. If an error was observed after 5 s, 1 point was added for each error operation, but if multiple errors occurred simultaneously, they were treated as 1 point [35]. The lower the error score, the better, and each action score and the total were calculated. There are five BESS items for error scoring: (1) raising the hand from the iliac crest; (2) opening your eyes; (3) any step, stumble, or fall; (4) the hip joint moved to over a 30-degree abduction; and (5) lifting the forefoot or heel [35]. The BESS results between repeat assessments by the same rater were excellent (reliability: 0.980).

The SEBT was calculated by standardizing the distance reached in each direction by the leg length (from the anterior iliac spine to the medial malleolus). The difference in reaching distance between both sides was the difference in distance from the dominant to the nondominant and was presented as an absolute value (Equation (1)) [31]. The length of the lower extremity and the distance to reach it were calculated by the same rater using the ratio method of Kinovea software (reliability: 0.917).

(1) Differences in reaching distance = |Dominant leg−Nondominant leg|

The incidence of injured musculoskeletal injuries in 77 participants collected over a 6-month period was calculated by frequency, type, and cause of injury and recovery period.

### 2.4. Statistical Analysis

Spearman’s rank correlation analysis was performed for confirming the applicability of SAC to the evaluation tool for postural control of lower extremities. Binary logistic regression was performed to estimate the causal relationship between injury occurrence and SAC, injury occurrence, and postural control evaluation tools, respectively. An independent *t*-test and Mann–Whitney *U*-test were used to verify the difference in SAC score between the groups according to the presence or absence of lower limb damage and the postural control of the lower limb. SPSS 25.0 (IBM Corp., Armonk, NY, USA) was used for all statistical analysis, and the statistical significance level was set to α = 0.05.

## 3. Results

### 3.1. The Correlation between SAC and Evaluation Tool for Postural Control of Lower Limb

Table 1 shows the correlation between the SAC score and the normalized reach distance of the SEBT’s dominant and nondominant legs. A positive correlation was observed between the immediate memory score of SAC and the normalized reach distances of SEBT (P and PL of the dominant leg, and PM and P of the nondominant leg, respectively). A positive correlation was observed between the delayed memory and the normalized reach distance of the SEBT (PM, P, PL, and L of the dominant leg, and M, PM, P, and PL of the nondominant leg, respectively). Among the total scores of SAC and normalized P and PL reach the distance of SEBT of the dominant leg, a positive correlation was observed. The negative correlations were also observed between SAC (delayed memory and total score) and the M-direction difference between the dominant and nondominant legs (Table 2). On the other hand, no correlation was observed between SAC results compared with LESS, BESS, and SEBT results (*p* > 0.05).

### 3.2. Injury History for 6 Months after Testing

A total of 14 cases of acute musculoskeletal injury were reported in 77 participants collected over 6 months. Participants were classified into injured (*n* = 11) and healthy (*n* = 66) groups. One participant reported injuries to the ankle, lower leg, and hip, respectively, and the other participant reported injuries to the ankle and lower leg, respectively. The injured body parts were the ankle (*n* = 5, 35.7%), foot (*n* = 1, 7.1%), lower leg (*n* = 2, 14.3%), knee (*n* = 2, 14.3%), thigh (*n* = 2, 14.3%), and hip (*n* = 2, 14.3%). The types of injury included sprain (*n* = 4, 28.6%), strain (*n* = 3, 21.4%), bruise (*n* = 3, 21.4%), fracture (*n* = 1, 7.1%), ligament rupture (*n* = 1, 7.1%), cartilage injury (*n* = 1, 7.1%), and cramp (*n* = 1, 7.1%). The causes of injury were noncontact injury (*n* = 9, 64.3%), collision with other players (*n* = 4, 28.6%), and collision with moving objects (*n* = 1, 7.1%). The recovery period was 0 days (*n* = 6, 42.9%), 30 days or more (*n* = 5, 35.7%), 1 day (*n* = 1, 7.1%), 2 days (*n* = 1, 7.1%), and 7 days (*n* = 1, 7.1%).

### 3.3. Predicting Injury Occurrence

As a result of analyzing the accuracy of the classification of the injury occurrence group by logistic regression, the statistical significance of the individual independent variables for the presence or absence of injury was analyzed. Each result from logistic regression model suggests that the overall model was not found to be statistically significant (*p* > 0.05; Table 3). It was found that the independent variables (SAC, LESS, BESS, and SEBT) did not affect the presence or absence of injury (*p* > 0.05; Table 3).

### 3.4. Difference between Injured and Healthy Groups

Statistical differences between groups were not observed in the SAC score and lower limb function performance evaluation results (Table 4).

## 4. Discussion

This study aims to assess if neurocognitive assessment can identify risk factors of the lower extremity and to analyze the association between the neurocognitive level and the occurrence of acute musculoskeletal injuries in male collegiate athletes. The major findings of this study are twofold: first, SAC evaluating neurocognitive function of collegiate athletes and dynamic postural control have small to medium correlations, and second, however, lower extremity injuries cannot be predicted using the SAC. As a result of this study, the correlation between SAC results (immediate memory, delayed memory, and total score) and SEBT result was observed. This result was consistent with our hypothesis. Even though motor skills including gross control such as balance, walking, agility, and flexibility were weakly associated with neurocognitive skills, neurocognitive function has been reported to be associated with motor skills, which may support the result of this study. The strength of link between neurocognitive function and motor skill is influenced by the difficulty of the task [36,37,38]. Therefore, SEBT, which is dynamic postural assessment tool, can be a difficult motor skill (novelty, complex/difficult task) that can be affected by neurocognitive function [25,39,40,41]. Since SEBT is more goal directed motor action and need specific (high-order) neurocognitive control process, SEBT is more likely to be affected by neurocognitive level than other balance task. According to the results of a prospective cohort using SEBT, lower limb injuries were reported in high school basketball players with normalized A, PM, and PL reach of 94% or less [31]. In addition, a systematic review has reported that SEBT is associated with an increased risk of injury [42,43]. This study suggests the possibility of predicting lower extremity injuries through correlation between the memory area that is the sub-domain of the SAC and SEBT result.

Based on the results of this study, it was found that lower extremity injuries cannot be predicted using the SAC. This result was inconsistent with our hypothesis. In a study conducted on alpine skiers and snowboarders, there was no difference in neurocognitive scores between the injured and non-injured groups, which is similar to the results of the present study [44]. Although the neurocognitive evaluation was applied to those who did not have a history of concussion within the past six months when recruiting the participants, it is thought that the cumulative shock received by the subjects participating in each of their sports during the six months of data collection may have resulted in impairment that may have affected neurocognitive function. These effects would have decreased physical function and increased the risk of musculoskeletal injury and concussion [45]. Therefore, it is necessary to periodically record and observe SAC scores for athletes who participate in non-net sports.

The criteria for judging sports concussion during training and competition are classified by coaching staff in the field according to the athlete’s awareness and loss of consciousness. The understanding of both players and leaders regarding concussion was high, but there was a problem with the classification and management of the actual concussion [46,47]. The recovery period of a simple concussion is reported to occur immediately or within 10 days, depending on the degree [48,49]. However, some athletes do not fully recover from concussion and are more likely to be exposed to other injuries when returning to the field [11,50]. Athletes are reluctant to talk about their symptoms because of the fear of being excluded from the entry list. In the case of the subjects of this study, it is possible that less number of injuries was reported to the team trainer because they are selected to the professional team based on their grades on the university team.

In the SAC results of this study, no statistical difference was observed between the injured and non-injured groups. Ha reported that have no difference in scores of computerized neurocognitive tests between retired contact-sports athletes and control [51]. However, retired contact-sports athletes showed slower gait speeds during dual-task walking because the cognitive task is preceded any movement for walking [51]. A decline in neurocognitive ability may reduce the ability to cope with rapidly changing situations and increase the likelihood of injury. Previous studies have reported that cardiopulmonary training [52] and resistance training [53] improve memory and selective concentration, which are sub-domains of neurocognitive ability. Although athletes who participate in non-net sports repeatedly experience impacts on the head, it is thought that aerobic training and resistance training, in which the athletes regularly participated to improve physical ability, also influenced neurocognitive ability. Therefore, there might be difficulties in predicting injury through changes in neurocognition while participating in sports competitions and training. However, loss of proprioception information due to a past history of musculoskeletal injury and reduction in sports activities after retirements, such as understanding tactics and aerobic exercise, can cause a decrease in neurocognitive ability. 

Nevertheless, this study has some limitations. First, the injury follow-up period was as short as six months. A previous study reported that 6–12 months after concussion were greater incidence of the lower extremity injury than 0 to 3 and 3 to 6 months [11]. In future studies, the duration of injury follow-up should be considered. Second, the injured group that was investigated had a relatively small sample size, which is a usual limitation in prospective research. Therefore, further research is needed because the resulting analysis is subject to limitations. Third, baseball and basketball, which have relatively low concussion rates, were included in this study. However, traumatic brain injury has been an important issue in baseball [54]. Some concussed baseball players showed no symptoms when they returned to play, but residual effects on their batting technique were reported [55]. Cognition and perception are the most important factors in playing basketball [56]. If a head injury such as a concussion occurs, these can be affected. Unfortunately, basketball had the highest competition-related rates of concussion for partial-contact sports such as soccer [57]. Fourth, the SAC has so far been used as a cognitive evaluation tool in sports sites [23], but this method was designed about twenty years ago. Because there is a point of contention in the method, which was developed some time ago, further research will be required for the development of a new cognitive evaluation tool that is both valid and reliable. Lastly, follow-up data about the injury incidence were not collected in the same way because the trainer’s employment was different, which depended on the participants’ team circumstances.

## 5. Conclusions

The SAC score of college male non-net sports players alone was unable to predict the occurrence of injury. Therefore, using the SAC score alone to determine the risk factor of lower extremity injuries, except in the use of assessment after a concussion, should be cautioned against.

## Figures and Tables

**Table 1:** Correlation between neurocognitive testing score and normalized reaching distance in the Star Excursion Balance Test.

**Items**	**Dominant Leg**								
		**A**	**AM**	**M**	**PM**	**P**	**PL**	**L**	**AL**
Orientation	ρ	0.127	0.126	0.077	−0.078	0.023	0.030	0.086	0.099
	*p*	0.269	0.277	0.505	0.502	0.845	0.797	0.459	0.391
Immediate memory	ρ	−0.058	0.169	0.125	0.210	0.341	0.359	0.216	0.018
	*p*	0.617	0.142	0.280	0.067	0.002 **	0.001 **	0.059	0.876
Concentration	ρ	−0.253	−0.120	−0.192	−0.101	0.017	−0.060	0.009	−0.193
	*p*	0.026	0.299	0.094	0.384	0.883	0.604	0.935	0.093
Delayed memory	ρ	−0.029	0.243	0.194	0.318	0.397	0.414	0.336	−0.023
	*p*	0.799	0.033	0.091	0.005 **	<0.001 ***	<0.001 ***	0.003 **	0.846
Total score	ρ	−0.155	0.083	0.007	0.127	0.301	0.247	0.209	−0.119
	*p*	0.178	0.472	0.951	0.272	0.008 **	0.030 *	0.068	0.302
	**Nondominant Leg**								
Orientation	ρ	0.037	0.004	−0.086	−0.069	−0.112	0.082	0.171	0.151
	*p*	0.750	0.971	0.459	0.549	0.332	0.480	0.137	0.189
Immediate memory	ρ	0.161	0.050	−0.163	0.312	0.316	0.194	0.106	0.010
	*p*	0.162	0.668	0.158	0.006 **	0.005 **	0.090	0.357	0.928
Concentration	ρ	−0.137	−0.224	−0.102	−0.149	−0.110	−0.159	−0.125	−0.035
	*p*	0.233	0.051	0.379	0.196	0.340	0.168	0.279	0.762
Delayed memory	ρ	0.164	0.072	0.243	0.374	0.338	0.225	0.113	0.078
	*p*	0.154	0.533	0.034 *	0.001 **	0.003 **	0.049 *	0.326	0.498
Total score	ρ	0.054	−0.077	0.099	0.161	0.177	0.065	0.016	−0.013
	*p*	0.644	0.505	0.392	0.163	0.124	0.573	0.893	0.909

A, anterior; AL, anterolateral; AM, anteromedial; L, lateral; M, medial; P, posterior; PL, posterolateral; PM, posteromedial. * *p* < 0.05; ** *p* < 0.01; *** *p* < 0.001.

**Table 2:** The correlation between neurocognitive testing score and differences of reaching distance in the Star Excursion Balance Test.

Domain		A	AM	M	PM	P	PL	L	AL
Orientation	ρ	0.075	−0.133	0.006	−0.213	0.086	−0.077	0.060	−0.137
	*p*	0.519	0.249	0.961	0.063	0.459	0.507	0.603	0.236
Immediate memory	ρ	−0.175	−0.023	−0.186	0.189	0.006	0.045	0.020	0.019
	*p*	0.128	0.841	0.105	0.099	0.962	0.694	0.864	0.869
Concentration	ρ	−0.081	−0.094	−0.215	−0.069	0.217	0.025	0.106	−0.130
	*p*	0.483	0.415	0.060	0.552	0.058	0.831	0.357	0.261
Delayed memory	ρ	−0.042	−0.001	−0.229	0.024	0.145	0.076	0.075	−0.138
	*p*	0.716	0.996	0.045 *	0.838	0.209	0.511	0.518	0.231
Total score	ρ	−0.150	−0.034	−0.253	0.050	0.199	0.054	0.123	−0.169
	*p*	0.194	0.770	0.027 *	0.669	0.083	0.640	0.287	0.143

A, anterior; AL, anterolateral; AM, anteromedial; L, lateral; M, medial; P, posterior; PL, posterolateral; PM, posteromedial. * *p* < 0.05.

**Table 3:** Final logistic regression results for the association of the variables with injuries.

Domain	β	*p* Value	OR	95% CI for OR
**SAC**				
Orientation	0.840	0.272	2.317	0.518 to 10.366
Immediate memory	0.273	0.502	1.314	0.592 to 2.920
Concentration	0.051	0.814	1.052	0.687 to 1.613
Delayed memory	0.305	0.426	1.356	0.641 to 2.871
Constant	−9.289	0.049	<0.001	
−2 Loglikelihood = 59.442, χ^2^ = 5.083 (df = 4, *p* = 0.279), Nagerkerke *R*^2^ = 0.114				
**LESS**	−0.022	0.923	0.978	0.628 to 1.524
Constant	−1.694	0.110	0.184	
−2 Loglikelihood = 63.149, χ^2^ = 0.009 (df = 1, *p* = 0.923), Nagerkerke *R*^2^ < 0.001				
**BESS**				
Single-leg stance	−0.061	0.540	0.941	0.775 to 1.143
Tandem stance	−0.003	0.980	0.997	0.779 to 1.276
Double-leg stance on foam	0.392	0.219	1.480	0.792 to 2.766
Single-leg stance on foam	15.772	0.998	7,071,554.032	-
Tandem stance on foam	0.283	0.301	1.327	0.776 to 2.270
Constant	−161.821	0.998	<0.001	
−2 Loglikelihood = 57.640, χ^2^ = 5.517 (df = 5, *p* = 0.356), Nagerkerke *R*^2^ = 0.124				
**Normalized reaching distance of dominant leg in SEBT**				
Anterior	−0.023	0.690	0.977	0.871 to 1.096
Anteromedial	0.019	0.803	1.019	0.880 to 1.179
Medial	−0.018	0.492	0.982	0.934 to 1.033
Posteromedial	−0.049	0.293	0.952	0.869 to 1.043
Posterior	0.006	0.848	1.006	0.947 to 1.068
Posterolateral	0.079	0.098	1.082	0.985 to 1.189
Lateral	−0.020	0.652	0.980	0.898 to 1.070
Anterolateral	<0.001	0.997	1.000	0.934 to 1.071
Constant	−1.321	0.750	0.267	
−2 Loglikelihood = 59.442, χ^2^ = 3.716 (df = 8, *p* = 0.882), Nagerkerke *R*^2^ = 0.084				
**Normalized reaching distance of nondominant leg in SEBT**				
Anterior	0.002	0.966	1.002	0.897 to 1.120
Anteromedial	−0.021	0.752	0.979	0.860 to 1.115
Medial	−0.021	0.745	0.979	0.864 to 1.110
Posteromedial	0.034	0.484	1.035	0.940 to 1.139
Posterior	−0.009	0.839	0.991	0.906 to 1.083
Posterolateral	0.018	0.699	1.018	0.929 to 1.116
Lateral	−0.041	0.357	0.960	0.546 to 1.048
Anterolateral	0.031	0.540	1.031	0.935 to 1.138
Constant	−1.485	0.635	0.226	
−2 Loglikelihood = 61.393, χ^2^ = 1.765 (df = 8, *p* = 0.987), Nagerkerke *R*^2^ = 0.040				
**Differences of reaching distance in SEBT**				
Anterior	−0.014	0.879	0.986	0.826 to 1.178
Anteromedial	0.051	0.595	1.052	0.872 to 1.270
Medial	−0.073	0.407	0.930	0.783 to 1.104
Posteromedial	<0.001	0.998	1.000	0.870 to 1.149
Posterior	−0.040	0.558	0.961	0.842 to 1.097
Posterolateral	−0.003	0.963	0.997	0.873 to 1.138
Lateral	−0.107	0.235	0.898	0.753 to 1.072
Anterolateral	0.019	0.813	1.019	0.874 to 1.188
Constant	−0.821	0.455	0.440	
−2 Loglikelihood = 59.165, χ^2^ = 3.993 (df = 8, *p* = 0.858), Nagerkerke *R*^2^ = 0.090				

BESS, Balance Error Scoring System; CI, confidence interval; df, degree of freedom; LESS, Landing Error Scoring System; OR, odds ratio; SAC, Standardized Assessment of Concussion; SEBT, Star Excursion Balance Test.

**Table 4:** The results of the independent *t*-test between non-injured and injured groups.

Domain	Non-Injured (*n* = 66)	Injured (*n* = 11)	Z *t* (df) §	*p* Value
**SAC**				
Orientation	4.53 ± 0.56	4.73 ± 0.47	1.074	0.283
Immediate memory	5.70 ± 1.21	6.27 ± 1.19	1.441	0.150
Concentration	7.89 ± 1.70	8.27 ± 1.27	0.550	0.583
Delayed memory	4.70 ± 1.14	5.45 ± 1.57	1.524	0.127
Total score	22.82 ± 3.05	24.73 ± 3.07	1.923 (75) §	0.058
**LESS**				
Total error of LESS	4.50 ± 1.38	4.45 ± 1.86	0.447	0.655
**BESS**				
Double-leg stance	0.00 ± 0.00	0.00 ± 0.00		
Single-leg stance	6.53 ± 3.50	6.00 ± 3.46	−0.536	0.592
Tandem stance	2.29 ± 2.68	2.73 ± 3.00	0.657	0.511
Double-leg stance on foam	0.23 ± 0.65	0.64 ± 1.43	0.586	0.558
Single-leg stance on foam	9.70 ±1.35	10.00 ± 0.00	1.034	0.301
Tandem stance on foam	8.67 ± 2.45	9.64 ± 0.92	0.956	0.339
Total error of BESS	27.41 ± 6.25	29.00 ± 5.78	0.789 (75) §	0.433
**Normalized reaching distance of dominant leg in SEBT**				
Anterior	80.77 ± 10.35	79.05 ± 6.42	0.533 (75) §	0.596
Anteromedial	86.65 ± 12.28	85.39 ± 6.48	0.262	0.793
Medial	88.20 ± 15.64	85.95 ± 8.36	−0.742	0.458
Posteromedial	94.66 ± 13.91	93.28 ± 10.14	0.316 (75) §	0.753
Posterior	86.90 ± 14.29	88.14 ± 7.16	−0.282 (75) §	0.779
Posterolateral	86.09 ± 14.51	89.73 ± 8.99	1.121	0.262
Lateral	75.30 ± 15.23	76.34 ± 8.26	<0.001	1.000
Anterolateral	72.68 ± 12.93	72.60 ± 11.28	0.020 (75) §	0.984
**Normalized reaching distance of nondominant leg in SEBT**				
Anterior	80.94 ± 11.48	80.42 ± 6.85	0.102	0.919
Anteromedial	88.37 ± 11.77	86.80 ± 8.04	0.425 (75) §	0.672
Medial	90.10 ± 12.29	88.94 ± 7.59	−0.480	0.631
Posteromedial	96.45 ± 14.76	97.12 ± 11.51	0.269	0.788
Posterior	88.24 ± 14.90	87.29 ± 6.21	0.335	0.738
Posterolateral	83.43 ± 14.18	83.37 ± 6.02	0.015 (75) §	0.988
Lateral	73.99 ± 14.31	71.53 ± 8.54	0.552 (75) §	0.582
Anterolateral	71.05 ± 11.46	71.15 ± 8.54	−0.027 (75) §	0.978
**Differences of reaching distance in SEBT**				
Anterior	4.70 ± 3.95	4.12 ± 3.86	−0.655	0.512
Anteromedial	4.83 ± 3.85	5.41 ± 4.48	0.291	0.771
Medial	7.52 ± 10.70	5.40 ± 3.33	−0.524	0.600
Posteromedial	6.40 ± 5.58	6.53 ± 4.12	0.590	0.555
Posterior	6.06 ± 9.80	4.54 ± 2.90	0.029	0.977
Posterolateral	7.53 ± 6.04	6.87 ± 5.43	−0.175	0.861
Lateral	6.87 ± 5.00	4.73 ± 3.71	−1.310	0.190
Anterolateral	5.19 ± 4.76	6.68 ± 2.77	1.791	0.073

BESS, Balance Error Scoring System; df, degree of freedom; LESS, Landing Error Scoring System; M, mean; SAC, Standardized Assessment of Concussion; SD, standard deviation; SEBT, Star Excursion Balance Test; §, Independent *t*-test value. Values are expressed as mean ± standard deviation.
